# Pathogenicity of Bovine Neonatal Pancytopenia-associated vaccine-induced alloantibodies correlates with Major Histocompatibility Complex class I expression

**DOI:** 10.1038/srep12748

**Published:** 2015-08-03

**Authors:** Lindert Benedictus, Rutger D. Luteijn, Henny Otten, Robert Jan Lebbink, Peter J. S. van Kooten, Emmanuel J. H. J. Wiertz, Victor P. M. G. Rutten, Ad P. Koets

**Affiliations:** 1 Department of Infectious Diseases and Immunology, Faculty of Veterinary Medicine, Utrecht University, Utrecht, The Netherlands; 2 Department of Medical Microbiology, University Medical Center Utrecht, Utrecht, The Netherlands; 3 Laboratory of Translational Immunology, University Medical Center Utrecht, Utrecht, The Netherlands; 4 Department of Veterinary Tropical Diseases, Faculty of Veterinary Science, University of Pretoria, Onderstepoort, South Africa; 5 Department of Farm Animal Health, Faculty of Veterinary Medicine, Utrecht University, Utrecht, The Netherlands

## Abstract

Bovine Neonatal Pancytopenia (BNP), a fatal bleeding syndrome of neonatal calves, is caused by maternal alloantibodies absorbed from colostrum and is characterized by lymphocytopenia, thrombocytopenia and bone marrow hypoplasia. An inactivated viral vaccine is the likely source of alloantigens inducing BNP-associated alloantibodies in the dam. In this study the specificity of BNP alloantibodies was assessed and was linked to the pathology of BNP. We demonstrated that Major Histocompatibility Complex class I (MHC I) and Very Late Antigen-3, an integrin α3/β1 heterodimer, were the major targets of BNP alloantibodies. However, alloantibody binding to various bovine cell types correlated with MHC I expression, rather than integrin β1 or α3 expression. Likewise, alloantibody-dependent complement-mediated cell lysis correlated strongly with MHC I expression. Examination of several tissues of third trimester bovine foetuses revealed that cells, shown to be affected in calves with BNP, were characterized by high MHC class I expression and high levels of alloantibody binding. We conclude that in spite of the heterogeneous specificity of BNP associated maternal alloantibodies, MHC I-specific antibodies mediate the pathogenicity of BNP in the calf and that cells with high MHC I expression were preferentially affected in BNP.

Bovine Neonatal Pancytopenia (BNP), a fatal bleeding syndrome in neonatal calves, is characterized by lymphocytopenia, thrombocytopenia, bone marrow hypoplasia and severe internal and external bleeding^1^,^2^,^3^. BNP was first seen in 2006 and quickly emerged all over Europe^3^,^4^,^5^. Epidemiological studies showed a strong association between the occurrence of BNP and vaccination of the mothers of affected calves with Pregsure© BVD (Pfizer Animal Health)^5^. BNP has been reproduced in calves by feeding colostrum from dams that had previously given birth to calves that succumbed to BNP^1^,^6^ and several studies have shown the presence of alloantibodies recognizing calf leukocytes in the colostrum of these cows^7^,^8^,^9^. Experimental immunization of calves with PregSure© BVD induced alloantibodies that recognize the cell line used for the production of the vaccine^8^,^10^. Bovine Major Histocompatibility Complex class I (MHC I) proteins were shown to be present in the PregSure© BVD vaccine and were a target of BNP-associated alloantibodies^9^,^11^. Therefore, it is likely that vaccination of dams with Pregsure© BVD induced alloantibodies which, upon ingestion of colostrum, elicited BNP in calves.

In a recent study, we showed that there was no association between the occurrence of BNP and MHC I haplotypes of dams or calves^12^. MHC I is expressed on all nucleated cells, whereas BNP pathology is characterized by a loss of specific cell types (i.e. leukocytes, platelets and bone marrow cells)^1^,^2^,^3^. Some authors have therefore argued that alloantibodies with a different specificity than MHC I might better explain the pathogenesis of BNP^7^,^13^,^14^. The goals of the present study were to i) assess the relative importance of anti-MHC I antibodies, ii) elucidate if BNP-associated alloantibodies recognize other targets, and iii) link the alloantibody specificity to the pathology of Bovine Neonatal Pancytopenia.

## Results

### BNP-associated alloantibodies recognize MHC class I and bind cells with a diverse MHC class I background

**Figure 1:**
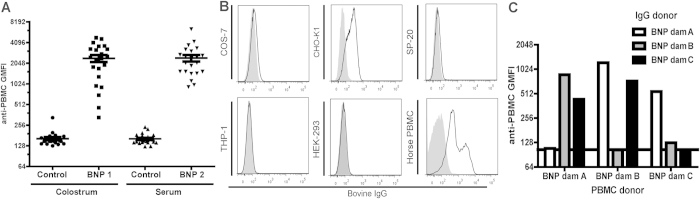
BNP alloantibodies recognize cells from a diverse MHC class I background. (**a**) Bovine PBMC isolated from non-BNP (n = 12) and BNP (n = 10) dams were stained with IgG isolated from the colostrum or serum of two dams. PBMC and IgG donors were MHC class I haplotyped and haplotyping results are listed online in Supplementary Table SI. (**b**) Cells from different species were stained with IgG isolated from BNP dams. Data are representative for three different BNP Ab donors. COS-7 = African green monkey. CHO-K1 = Chinese hamster. SP-20 = Mouse. THP-1 = Human. HEK-293 = Human. Live cells were gated on forward and sideward scatter. (**c**) PBMC isolated from three dams were cross-stained with IgG isolated from the same animals to test whether alloantibodies recognize autoantigens. The horizontal dotted line depicts the average GMFI of PBMC staining by autologous IgG. In all experiments Ab staining was measured using flow cytometry. GMFI = Geometric Mean Fluorescent Intensity.

First, we tested the specificity of BNP-associated alloantibodies and assessed how frequently alloepitope (mis)matches between bovine cell donors, vaccine and BNP dams occur. PBMC isolated from non-BNP and BNP dams were stained with IgG isolated from the serum or colostrum of two different BNP dams. Figure 1a shows the broad recognition and almost equal staining of PBMC from different donors by BNP alloantibodies, despite the diverse MHC I background of cell donors (see Supplementary Table SI online). There was no difference in staining of PBMC isolated from BNP or non-BNP dams. To further test the specificity of BNP alloantibodies, cell lines from different species were stained with IgG isolated from BNP dams (Fig. 1b). BNP alloantibodies reacted with Cho-K1 cells (Chinese Hamster) and Horse PBMC, showing that BNP alloantibodies recognized targets across species. BNP alloantibodies did not bind self PBMC (Fig. 1c). This confirms that tolerance to autoantigens was not broken and that there is a “gap” in the repertoire of BNP alloantibodies for self MHC I.

**Figure 2:**
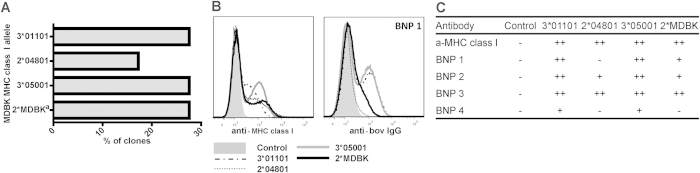
BNP alloantibodies recognize MHC class I alleles carried by the cell line used for vaccine production. (**a**) 29 full length MHC class I clones derived from MDBK cells, the cell line used for the production of Pregsure© BVD, were sequenced and the relative number of clones per identified allele are depicted. (**b**,**c**) Full length MDBK-derived MHC class I clones or a mock construct were transfected into HEK293 cells, stained with anti-bovine MHC class I mAb ILA88 as a positive control and IgG isolated from the serum of one (**b**) or four (**c**) BNP dam(s) and Ab binding was revealed using flow cytometry. BNP 1 in c is the same dam as in b. Results are based on two separate transfection experiments. ^a^Denotes a local name (Genbank accession number KM397368).

Since BNP alloantibodies recognized cells with very diverse MHC I backgrounds, we examined the specificity of BNP Abs for MHC I alleles of the cell line used for the production of the Pregsure© BVD vaccine. Full-length MHC I alleles from this Madin Darby Bovine Kidney (MDBK) cell line were cloned and expressed in HEK-293 cells. Bovine cells co-dominantly express between 2–6 classical MHC I alleles^15^ and sequencing of the MHC I clones identified four classical MHC I alleles transcribed at equal levels in the MDBK cells (Fig. 2a). Staining of MHC I transfected HEK-293 cells with Abs isolated from BNP dams revealed that Abs from all dams recognized MHC I. However, the recognition of specific MDBK-derived MHC I alleles was different between Abs isolated from different dams (Fig. 2b,c). We investigated if there was a common sequence or (linear) protein motif in MHC I alleles recognised by BNP alloantibodies which could explain the broad MHC I cross-reactivity of the alloantibodies. However, phylogenetic trees and protein alignments revealed no apparent relation between differences in MHC class I protein sequences and BNP alloantibody binding to specific MHC I alleles (see Supplementary Figure S1).

### BNP-associated alloantibodies recognize Integrin beta-1, alpha-3 and MHC I, but the majority of Abs have MHC I specificity

**Figure 3:**
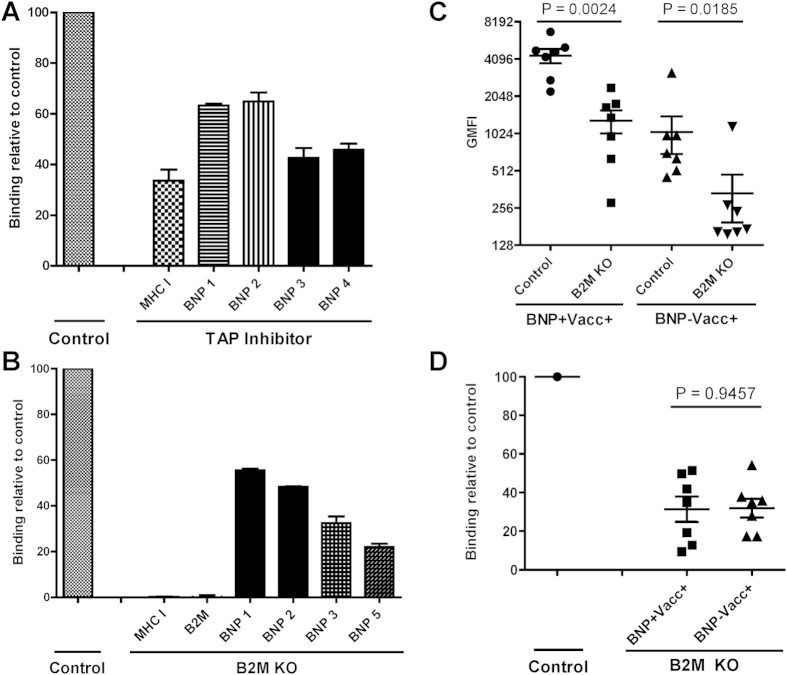
MHC class I is a major, but not the only, target of BNP-associated alloantibodies. (**a**) Comparison of MHC class I expression and binding of IgG isolated from BNP dams between MDBK cells transduced with a viral TAP inhibitor and MDBK cells that were transduced with a control vector. Data were expressed relative to binding of the control cell line and are average of three separate experiments. MHC class I expression was measured using mAb ILA88. (**b**) Comparison of MHC class I and B2M expression and binding of IgG isolated from BNP dams between wild-type MDBK cells (Control) and B2M KO MDBK cells. Data expressed as in A. B2M expression was measured using mAb B1.1G6. (**c**) Wild-type MDBK cells (Control) and B2M KO MDBK cells were stained with sera from Pregsure© BVD-vaccinated BNP (BNP+Vacc+, n = 7) and non-BNP (BNP−Vacc+, n = 7) dams. Alloantibody binding on wild-type and B2M KO MDBK cells was compared using a paired t-test. (**d**) Data from c were transformed to express Ab binding to B2M KO MDBK relative to wild-type MDBK and relative alloantibody binding was compared using an unpaired t-test for equal variance. In all experiments Ab binding was measured using flow cytometry and respective isotype controls were subtracted. GMFI = Geometric mean fluorescent Intensity.

TAP inhibition leads to MHC I down-regulation and to assess the proportion of BNP alloantibodies that recognize MHC I, we used MDBK cells expressing the Bovine Herpes Virus-1-derived TAP inhibitor UL49.5^16^. Both MHC I expression and BNP Ab binding were reduced on MDBK cells transduced with the viral TAP inhibitor (Fig. 3a), again confirming that BNP alloantibodies recognized MHC I. However, the reduction in BNP Ab binding was lower than the MHC I down regulation, indicating that BNP alloantibodies also recognized non-MHC I targets.

Knocking-out B2M leads to retention of MHC I in the ER and almost completely abolishes MHC I expression at the cell surface^17^. To confirm that BNP alloantibodies recognize non-MHC I targets we constructed a B2M knockout MDBK cell line (B2M KO) using the CRISPR/Cas9 gene editing technique. Using flow cytometry we confirmed that there was no residual B2M and MHC I expression on the B2M KO cell line (Fig. 3b). There was a marked reduction in binding of IgG isolated from serum of BNP dams to B2M KO cells compared to wild-type MDBK cells (Fig. 3b). Staining cells with serum from Pregsure© BVD-vaccinated BNP and non-BNP dams showed a significant reduction in Ab binding on the B2M KO cells for both groups (Fig. 3c). The relative binding of B2M KO cells was similar between Pregsure© BVD-vaccinated BNP and non-BNP dams and ranged between 9–54% (Fig. 3d). These results demonstrated that MHC I is the major target of BNP-associated alloantibodies and depending on the BNP dam accounts for 46–91% of the Ab specificity. Conversely, 9–54% of the Abs were specific for non-MHC I targets.

**Figure 4:**
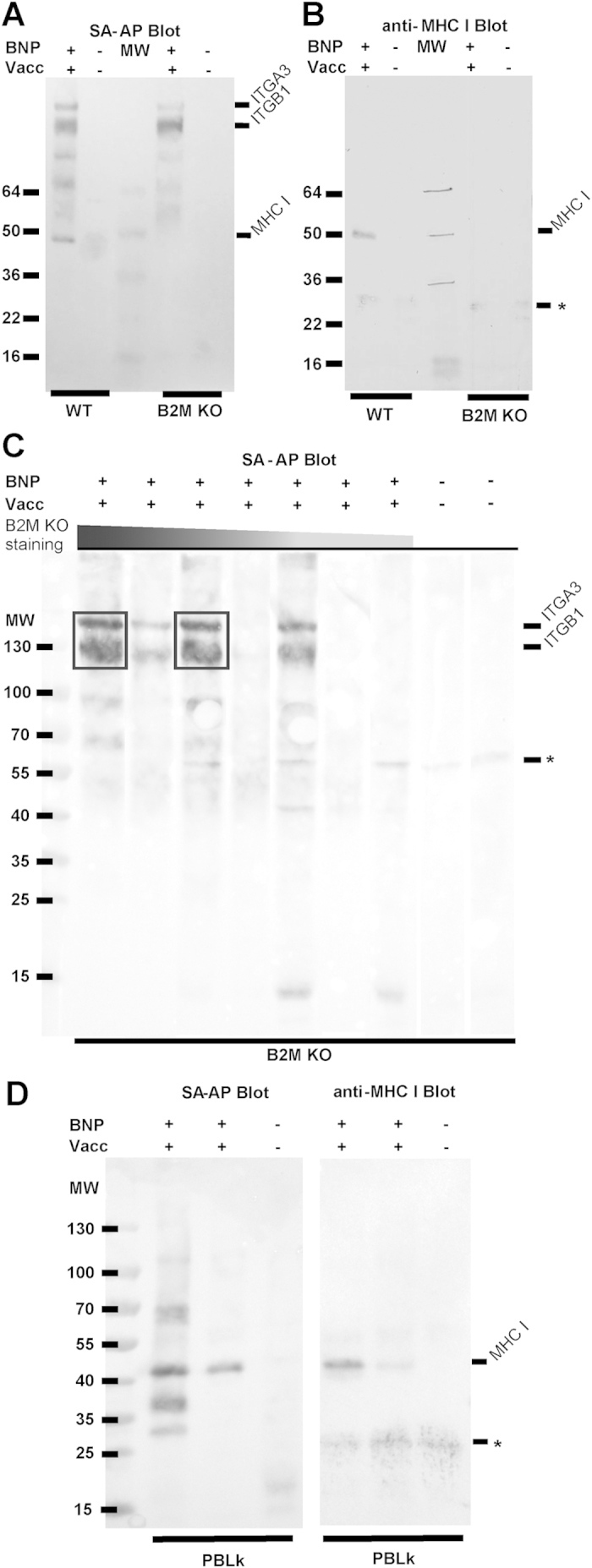
Sera from BNP dams recognize MHC class I and non-MHC class I targets. Cell surface proteins of wild-type MDBK (WT), B2M KO MDBK (B2M KO) or PBLk were biotinylated and subsequently stained with sera of Pregsure© BVD-vaccinated BNP dams (BNP+Vacc+) or control dams not vaccinated with Pregsure© BVD (BNP−Vacc−). After washing away unbound Abs, cells were lysed. Abs and bound Ag were precipitated using Protein G coupled dynabeads, separated by non-reducing gel electrophoresis and blotted on nitrocellulose membrane. (**a**) WT and KO MDBK cells were stained with serum from a BNP+Vacc+ dam or a BNP−Vacc− dam. Cell surface proteins were visualized using SA-AP. (**b**) As in A, but immunoprecipitated MHC class I was visualized using anti-bovine MHC class I mAb ILA88. (**c**) B2M KO MDBK cells were stained with sera from BNP+Vacc+ dams (n = 7) and BNP−Vacc− dams (n = 2). Cell surface proteins were visualized using SA-AP. The sera from BNP+Vacc+ dams were ranked according to alloantibody staining intensity of B2M KO MDBK cells as in Fig. 3c. Representative for two separate experiments. (**d**) PBLk from a healthy donor were stained with sera from BNP+Vacc+ dams (n = 2) or a BNP−Vacc− dam. Cell surface proteins and MHC I were visualized using SA-AP and mAb ILA88, respectively. Representative for two separate experiments with different PBLk donors. MW = Molecular weight markers (kDa). ITGB1 = integrin β1. ITGA3 = integrin α3. * = nonspecific signal. Boxes in C indicate regions that were subjected to mass spectrometry analysis.

To identify the non-MHC I targets of BNP alloantibodies, we immunoprecipitated target Ags on wild-type and B2M KO MDBK cells using sera from BNP dams. In B2M KO cells, MHC I is still expressed intracellularly (data not shown) and therefore care was taken to precipitate extracellular proteins only. Western blot analysis of antigens precipitated from wild-type MDBK cells using sera from BNP dams showed three prominent bands around 40, 130 and 140 kDa. The band around 40 kDa disappeared when using B2M KO cells and staining with an MHC I mAb confirmed that these precipitated proteins were MHC I (Fig. 4b). Results were similar when using serum from a Pregsure©-BVD vaccinated non-BNP dam, although the bands of precipitated proteins were less intense (data not shown). To see if sera from different dams precipitate similar Ags, we repeated the above experiment with sera from several BNP dams using B2M KO cells to focus on non-MHC I targets (Fig. 4c). Two prominent bands around 130 and 140 kDa were observed in the lanes of four of the seven BNP sera. To identify these non-MHC I targets of BNP alloantibodies, the precipitated antigens were subjected to mass spectrometry analysis. Two proteins were identified, Integrin alpha-3 and Integrin beta-1, that together form the receptor Very Late Antigen-3 (VLA-3) (Table 1).

### Binding of BNP Abs to various cell types and BNP antibody-dependent complement-mediated cell lysis correlates with MHC I expression, not Integrin beta-1 expression.

**Figure 5:**
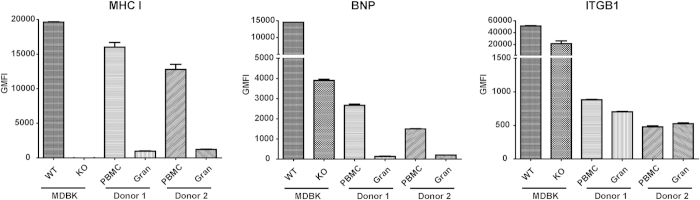
Expression of MHC class I, Integrin beta-1 and BNP Ab binding on wild-type (WT) and B2M knockout (KO) MDBK cells and PBLk. WT and B2M KO MDBK cells and PBLk (n = 2, donor 1 & 2) were stained with mAbs against MHC class I (MHC I), Integrin beta-1 (ITGB1) or with BNP Abs. Expression/Ab binding was measured using flow cytometry. GMFI = Geometric Mean Fluorescent Intensity.

We compared the degree of MHC I and integrin beta-1 expression to the level of BNP Ab binding of wild-type and B2M KO MDBK cells, peripheral blood leukocytes (PBLk) and platelets (Fig. 5 and Supplementary Figure S2 online). MHC I expression was high on wild-type MDBK cells and on PBMC, but was low on granulocytes, very low on platelets and absent on B2M KO MDBK cells. Integrin beta-1 expression showed a different pattern, with comparable expression levels on PBMC and granulocytes, intermediate expression on platelets and very high expression on both B2M KO and wild-type MDBK cells. BNP Ab binding on PBLk and platelets correlated with MHC I expression, rather than integrin beta-1 expression. We examined target Ags of BNP Abs on PBLk by immunoprecipitation. As shown in Fig. 4d, MHC I was immunoprecipitated on PBLk, whereas integrin beta-1 and alpha-3 were not, indicating BNP Abs only bind MHC I on PBLk.

**Figure 6:**
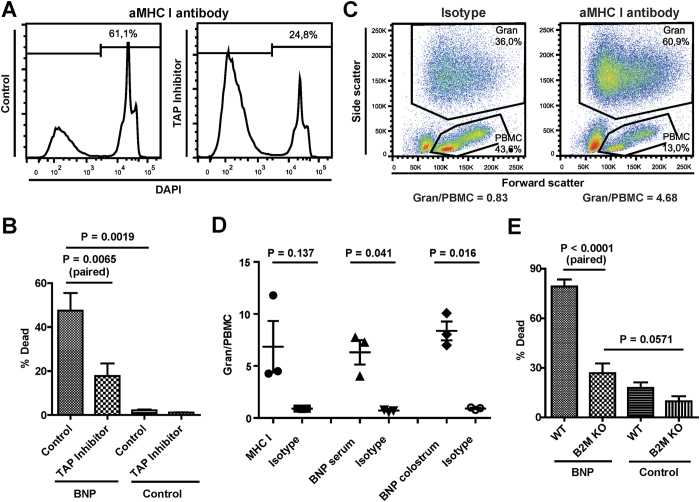
BNP antibody-dependent complement-mediated lysis of cells correlates with MHC class I expression. Cells were stained with antibodies and subsequently incubated with rabbit complement for 30 minutes. Antibody-dependent complement-mediated killing was measured as the percentage DAPI positive cells using flow cytometry. (**a**) MDBK cells transduced with the viral TAP inhibitor UL49.5 (TAP inhibitor) and MDBK cells that were transduced with a control vector (Control) were stained with an anti-MHC class I mAb (PT85a). (**b**) TAP inhibitor and control cells were stained with sera from Pregsure© BVD-vaccinated BNP dams (BNP, n = 6) and with unvaccinated control sera (control, n = 4). The difference in complement-mediated killing of Control and TAP inhibitor MDBK cells by sera from BNP dams and of control MDBK cells between BNP and control sera was compared using a paired t-test and an unpaired t-test for unequal variance, respectively. (**c**) Peripheral blood leukocytes (PBLk) were stained with anti-MHC class I mAb (ILA88) or isotype control. The proportion of granulocytes (Gran) to PBMC is given. (**d**) PBLk from calves (<6 mnd, n = 3) were stained with Abs from different sources. MHC I, Anti-MHC class I mAb and isotype control. BNP serum (/BNP colostrum), serum (/colostrum) from a Pregsure© BVD-vaccinated BNP dam and serum (/colostrum) from an unvaccinated dam as isotype control. The proportion of Gran to PBMC between Ab and isotype control is compared using a paired t-test. Data are representative for several different BNP sera and BNP colostra. (**e**) Wild-type (WT) and B2M knockout (B2M KO) MDBK cells were stained with sera from Pregsure© BVD-vaccinated BNP dams (BNP, n = 6) and unvaccinated control sera (control, n = 4). The difference in complement-mediated killing of WT and B2M KO MDBK cells by sera from BNP dams and of B2M KO MDBK cells between BNP sera and control sera was compared using a paired t-test and an unpaired t-test for equal variance, respectively.

All nucleated cell express MHC I, but as shown in Fig. 5 the expression levels of MHC I can differ greatly between cell types. We therefore investigated whether MHC I expression levels had implications for the pathogenic effects of BNP-associated alloantibodies. A recent study showed that BNP alloantibodies could induce complement-mediated lysis of MDBK cells^18^. MHC I mAb dependent complement-mediated cell lysis was lower in TAP-inhibited than in control MDBK cells (Fig. 6a), correlating with MHC I expression as found in Fig. 3a. Next, the same assay was performed using sera from several BNP dams (Fig. 6b). Complement-mediated cell lysis was significantly lower for TAP-inhibited MDBK cells compared to the control cell line (p = 0.0019), indicating that BNP Ab dependent complement-mediated cell lysis correlated with MHC I expression. MHC I expression is higher on PBMC than on granulocytes and MHC I mAb dependent complement-mediated lysis of PBLk strongly increased the granulocyte/PBMC ratio (Fig. 6c,d), reflecting the killing of PBMC rather than granulocytes. Similarly, incubating PBLk with serum or colostrum from BNP dams significantly increased the granulocyte/PBMC ratio (Fig. 6d). Tracking cell numbers using beads confirmed that, despite low BNP alloantibody binding and low MHC I expression, granulocytes were not lysed after BNP Ab mediated complement dependent cell lysis of PBLk (see Supplementary Figure S3 online). Together these data show that BNP antibody-mediated complement dependent lysis strongly correlates with MHC I expression and that cells with high MHC I expression are killed predominantly. In line with this, BNP antibody-mediated complement dependent cell lysis of B2M KO MDBK cells was lower than that of wild type-MDBK cells (p < 0.001, Fig. 6e). Despite the lack of MHC I expression, complement lysis of B2M KO MDBK cells appeared to be higher for sera from BNP dams than for control sera. Although this effect was not statistically significant (p = 0.0571, Fig. 6e), this indicates that non-MHC I alloantibodies may also activate complement and could in theory lead to cell lysis *in vivo*.

### Cells affected in Bovine Neonatal Pancytopenia are characterized by high MHC class I expression

**Figure 7:**
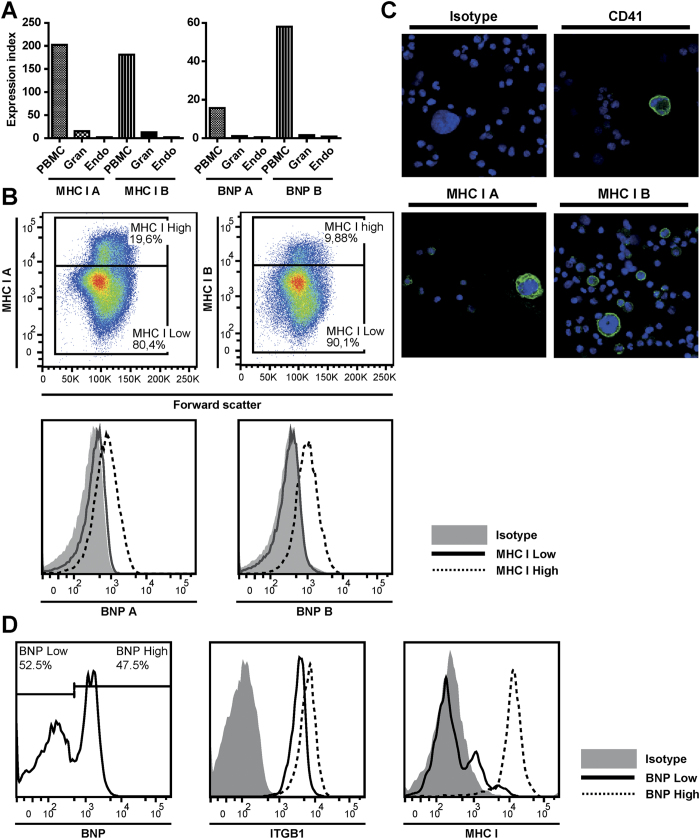
Cell types affected in Bovine Neonatal Pancytopenia are characterized by high MHC class I expression. (**a**–**c**) Bone marrow and endothelial cells were isolated from late gestation bovine foetuses (n = 3). MHC class I expression and BNP Ab binding was assessed using flow cytometry (a,b) and confocal imaging (**c**). Data are representative for three different animals. (**a**) Endothelial cells were mixed with peripheral blood leukocytes from a healthy donor to compare MHC class I expression and BNP Ab binding between tissues. Endothelial cells and PBMC/Granulocytes were selected on forward and sideward scatter (FSC and SSC) and on CD31^high^ and CD31^low^ expression, respectively. Expression index = Δ Ab/Δ Isotype. (**b**) MHC class I expression and BNP Ab binding of bone marrow cells. Bone marrow cells divided into MHC class I^high^ and MHC class I^low^ expression (upper panel) were compered for BNP alloantibody binding (lower panel). Cells were gated on live cells based on FSC and SSC (confirmed with DAPI) and on autofluorescent-negative cells. (**c**) Confocal images of bone marrow cells stained with isotype, anti-MHC class I or anti-CD41 (Green). DAPI (blue) was used as a nuclear stain. Megakaryocytes are cells with large nuclei and anti-CD41 was used as a positive control for megakaryocytes. (**d**) Cryopreserved bone marrow cells isolated from a neonatal calf were measured using flow cytometry to evaluate MHC class I and Integrin beta-1 expression of cells with low and high BNP Ab binding. Intact cells were gated based on FSC and SSC and dead cells were excluded using DAPI staining. Data are representative for Abs isolated from two different BNP dams.

After colostrum is taken up by the calf, maternal Abs are absorbed via intestinal cells and are transported throughout the body via the circulation. Indeed, alloantibodies were detected in the blood of BNP calves (data not shown). Nevertheless, only certain cell types are affected in BNP (i.e. lymphocytes, platelets and bone marrow cells), whereas endothelial cells which are in close contact with the absorbed alloantibodies are unaffected^1^,^6^. We investigated whether differential MHC I expression and BNP alloantibody binding of different cell types could explain these observations. Endothelial and bone marrow cells were isolated from third trimester foetal calves (7–9 months of gestation) and stained with MHC I mAbs and BNP alloantibodies (Fig. 7a–c). As shown in Fig. 7a, MHC I expression and BNP Ab binding was much lower on endothelial cells than on PBMC. Bone marrow cells were characterized by populations of high or low MHC I expression (Fig. 7b, upper panels) and these populations were also characterized by high or low BNP alloantibody binding, respectively (Fig. 7b, lower panels). Confocal images of bone marrow cells showed that megakaryocytes have high MHC I expression (Fig. 7c). MHC I bright and dim uni-nucleated cells, as seen with flow cytometry, were also observed on these images. Cryopreserved bone marrow cells isolated from a neonatal calf were divided into cells with high and low BNP Ab binding and we examined integrin beta-1 and MHC I expression in both populations (Fig. 7d). Although integrin beta-1 expression was somewhat lower in bone marrow cells with low BNP Ab binding, MHC I expression of bone marrow cells correlated much better to BNP Ab binding. Together these data showed that the cell types affected in BNP were characterized by high MHC I expression and high BNP alloantibody binding.

## Discussion

BNP is a fatal bleeding syndrome in neonatal calves caused by Pregsure© BVD vaccine-induced maternal alloantibodies absorbed from the colostrum. In this paper, we investigated the specificity of BNP-associated alloantibodies and linked the specificity of these Abs to the pathogenesis of BNP.

To investigate the importance of MHC I specific Abs in the pathogenesis of BNP, we assessed the relative quantity of MHC I specific BNP Abs. We generated a B2M knockout MDBK cell line using the CRISPR/Cas9 gene editing technique and showed that MHC I is a major target of BNP Abs (Fig. 3). The expression of MHC I-like molecules MR1 and CD1 also depends on B2M, but intra- and extracellular staining of MDBK cells with MR1 and CD1 specific Abs showed no expression of these proteins on MDBK cells (data not shown). Based on sequencing of the B2M gene, we previously concluded that it is highly unlikely that B2M itself is a target of BNP Abs^12^. Therefore, knocking out B2M only influences BNP Ab binding to MHC I. BNP Abs also recognized non-MHC I targets and mass spectrometry analysis of Ags immunoprecipitated using BNP alloantibodies revealed VLA-3 as an additional target of BNP Abs. VLA-3 is a heterodimer consisting of integrins β1 and α3 and for both these integrins many allelic variants are documented^19^.

BNP is characterized by the loss of specific cell types (i.e. lymphocytes, platelets and bone marrow cells), whereas other cells exposed to BNP Abs are not affected (e.g. endothelium). Bell *et al*.^1^ reproduced BNP in calves by feeding colostrum from BNP dams and found there was only a transient drop in neutrophil levels in blood, with levels restoring to normal within 12 hours after colostrum ingestion. Furthermore, they showed that in bone marrow, mature cells of the neutrophil, eosinophil and erythroid lineages were less affected, which was confirmed by other studies^9^,^20^. BNP Ab binding matched the pathology of BNP, with high Ab binding of cells that are affected in BNP such as PBMC and a subset of bone marrow cells, whereas endothelial cells and granulocytes showed very low BNP Ab binding (Figs 5 and 7). Although MHC I is expressed on all nucleated cells, the level of MHC I expression differed greatly between cell types and corresponded to BNP Ab binding (Figs 5 and 7). Interestingly, megakaryocytes, which are almost completely depleted in most BNP cases^1^,^3^, were also characterized by very high MHC I expression (Fig. 7). Following ingestion of colostrum from BNP dams, platelet counts in calves drop and remain low without an increase in reticulated thrombocytes^1^. This indicates that both direct depletion of platelets following BNP Ab binding (Supplementary Figure S2) and the depletion of megakaryocytes contributes to the low platelet counts in BNP calves. VLA-3 is typically expressed on endo- and epithelium^21^,^22^ and also on many cancer cells and immortalized cell lines^23^,^24^. However, BNP Abs marginally bound endothelial cells (Fig. 7) and epithelial and endothelial linings are not damaged in the course of BNP. Integrin β1 can associate with several alpha integrins and is expressed on virtually all cell types^25^. We confirmed the high expression of integrin β1 on MDBK cells (Fig. 5) and hence it is likely to be present in the Pregsure© BVD vaccine. The expression patterns of VLA-3 and integrin β1 did not match with the binding of BNP-associated alloantibodies and the pathology of BNP, whereas MHC I expression did. Furthermore, only four of seven sera from BNP dams clearly precipitated VLA-3 (Fig. 4c), indicating VLA-3 specificity is not a prerequisite for the development of BNP. The clinical and post mortem presentation of BNP is very consistent between cases^1^,^2^,^3^ and does not indicate a difference in clinically relevant alloantibody specificities between BNP dams. Therefore, we hypothesized MHC I specific alloantibodies drive the pathogenesis of BNP and susceptibility of cells to BNP-associated alloantibodies depends on MHC I expression levels.

A steep drop in lymphocyte and platelet counts can be seen within 2 hours after calves ingest colostrum from BNP dams^1^,^6^. This remarkably quick effect of BNP Abs suggests a pivotal role for antibody-dependent complement-mediated cell lysis of cells, rather than a cellular immune response^26^, in the pathogenesis of BNP. We showed that BNP Abs can activate complement and that subsequent cell lysis correlates with MHC I expression *in vitro* (Fig. 6). This corroborates our hypothesis that only cells with high MHC I expression are affected in BNP and is also in support of complement activation as an important effector mechanism of BNP alloantibodies *in vivo*.

Foetal/neonatal allo-immune thrombocytopenia (FNAIT) in humans is a syndrome caused by maternal anti-platelet alloantibodies and is characterized by a severe thrombocytopenia that can lead to life threatening haemorrhages^27^. FNAIT has been compared to BNP, but whereas FNAIT is only characterised by a thrombocytopenia, BNP is additionally characterized by lymphocytopenia and depletion of bone marrow cells. Alloantibodies in FNAIT are directed against platelet Ags, of which integrin β3 is most important. Paternal MHC I specific Abs can be found in 10–30% of pregnant women and have been associated with incidental cases of FNAIT^27^,^28^. Nevertheless, it is generally accepted that MHC I specific Abs do not play a (important) role in FNAIT^27^,^29^. In this respect FNAIT resembles our view of BNP; in both diseases alloantibodies with heterogeneous specificity may be present, but a dominant specificity (platelet antigens in FNAIT and MHC I in BNP) drives pathogenesis.

MHC I is expressed on the foetal membranes at the end of gestation^30^ and naturally occurring alloantibodies against paternal alloantigens can be detected in up to 64% of multiparous cattle^31^,^32^. The risk of the occurrence of BNP increases with parity^5^,^12^ and exposure to foetally expressed paternal MHC I could contribute to the Pregsure© BVD induced alloimmune response. Our data shows that each BNP dam has a specific alloantibody repertoire, which is expected since the alloantibody repertoire depends on the allogeneic background of the dam. However, we also show that BNP alloantibodies have a very broad specificity. The MHC I specificity of pregnancy-induced alloantibodies broadens with multiple gestations^31^,^33^ and this effect has also been seen after repeated vaccinations with allogeneic lymphocytes^31^. Through (multiple) Pregsure© BVD vaccination(s) and pregnancy, BNP dams are repeatedly exposed to alloantigens, which could explain the broad specificity of BNP alloantibodies we observed. We previously hypothesized that the quality of the alloantibody response is equal in Pregsure© BVD vaccinated non-BNP and BNP dams^12^. The finding that non-BNP dams have MHC I specific alloantibodies corroborates this hypothesis. The MHC I specificity of non-BNP dams has also been shown in a recent study by Kasonta *et al*.^18^. However, BNP dams have considerable higher alloantibody levels^8^,^12^. In humans, complement fixation correlates with alloantibody levels^34^ and high alloantibody levels are associated with increased risk of antibody-mediated rejection of allogeneic transplants^34^,^35^. Therefore, we hypothesized that the development of BNP in the calf primarily depends on the alloantibody dose the calf absorbs^12^. The odds of BNP increases with increased colostrum intake^5^ and as a corollary increased alloantibody intake, also indicating that the occurrence of BNP is alloantibody dose dependent. Incidental cases of calves with BNP symptoms without a history of Pregsure© BVD vaccination have been reported^1^,^36^,^37^. Although speculative, these cases could be related to pregnancy induced MHC I alloantibodies. Another explanation could be the use of other vaccines that contained bovine alloantigens. Two studies could not detect alloantibodies in dams vaccinated with other inactivated BVD vaccines^8^,^18^. However, Pregsure© BVD induced BVD antibody titres that were significantly higher than alternative BVD vaccines^8^,^38^ and this may have favoured the induction of high alloantibody levels. Following vaccination with different inactivated BVD vaccines, Bastian *et al*.^8^ detected low levels of antibodies that bound bovine leukocytes in the sera of guinea pigs and Deutskens *et al*.^11^ showed that the sera from some of these vaccinated dams precipitated antigens from MDBK cells, thus indicating that bovine proteins may also be present in other vaccines. In cows, maternal Abs are not transported across the placental barrier and are only absorbed from the colostrum in the first hours after birth. This sudden uptake of a large quantity of alloantibodies likely contributed to the sudden and severe presentation of BNP. In contrast, in humans Abs are transported across the placental barrier during pregnancy. Consequently, pathologic alloantibodies can already affect the human foetus during pregnancy and will likely lead to more chronic and less easily observed pathologies. In humans anti-paternal MHC I alloantibodies have been associated with pathology during pregnancy (e.g. chronic chorioamnionitis^39^ and recurrent miscarriage^40^), but results between studies are heterogeneous^41^. Influenza vaccination in humans has been shown to induce alloimmune responses^42^,^43^, but to our knowledge alloimmune related adverse effects of human vaccines produced on human cell lines have not been reported. Nevertheless, as adverse effects may be difficult to monitor, it remains prudent to be vigilant of possible alloimmune-related adverse effects of vaccines grown on same-species cell lines.

In this study we show MHC I and VLA-3 are major targets of BNP-associated vaccine-induced alloantibodies. However, antibody-dependent complement lysis assays showed that *in vitro* killing of cells correlates with MHC I expression and BNP Ab binding. Based on expression of target antigens and binding of BNP associated alloantibodies of tissues differentially affected in BNP, we conclude that MHC I-specific BNP alloantibodies mediate the pathogenicity of BNP in the calf and that cells with high MHC I expression were preferentially affected in BNP.

## Materials and Methods

### Animals and sample collection

Peripheral blood, serum and colostrum were collected from BNP, non-BNP and control dams. The following definitions were used to categorize dams:

- BNP dam, vaccinated with Pregsure© BVD and given birth to a calf which developed BNP, diagnosed based on clinical signs and hematology and/or pathology, following ingestion of the dam’s colostrum.
- Non-BNP dam, vaccinated with Pregsure© BVD and given birth to healthy calves that showed no clinical signs of BNP upon ingestion of the dam’s colostrum.
- Control dam, no Pregsure© BVD vaccination history and given birth to healthy calves.

IgG, purified from serum and delipidated colostrum by liquid affinity chromatography using HiTrap™ Protein G columns (GE Healthcare), was biotinylated with biotin-7-NHS (Roche) in a 1:20 molar ratio. Bovine and horse peripheral blood leukocytes (PBLk) were isolated by hypotonic lysis of erythrocytes^12^.

Non-bovine cell lines (COS-7, CHO-K1, SP-20, THP-1, HEK-293) used to detect BNP alloantibody binding across species were maintained as appropriate.

Late gestation bovine foetuses (n = 3) were collected at a commercial slaughterhouse and used to isolate bone marrow and endothelium. Gestation length, determined by measuring crown-rump length as described by Rexroad *et al*.^44^, was 7, 8, and 9 months(/a term) of gestation, respectively. Endothelial cells were isolated from the aorta by gently scraping the endothelial lining with a surgical blade, a method described by Ryan and Maxwell^45^. Both femurs were carefully opened to isolate bone marrow cells by rinsing the femur cortex with DMEM (Gibco) supplemented with 5 U/ml heparin. In addition, bone marrow cells isolated from a neonatal calf that died during caesarean section were cryopreserved in 10% DMSO at −80 °C before analysis.

This study was approved by the Animal Ethical Committee of Utrecht University and conducted according to their regulations.

### Sequence based MHC class I haplotyping

Sequence based MHC I haplotyping of non-BNP and BNP dams was performed as described by Benedictus *et al*.^12^. In short, gene-specific primers aligning with intron 1 and intron 3 of putative MHC I genes 1,2,3 and 6^46^ are used to amplify exons 2 and 3 encoding the most polymorphic region of the MHC I. PCR products were sequenced and SeqScape© (v2.5, Applied Biosystems) was used to match consensus sequences to a library of exon 2 and 3 sequences of all known MHC I alleles documented on the IPD MHC database (http://www.ebi.ac.uk/ipd/mhc/bola/). MHC I haplotypes were determined using haplotypes defined in Codner *et al*.^15^ and Benedictus *et al*.^12^.

### MDBK and MDBK-derived cell lines

Madin Darby Bovine Kidney cell line (MDBK; ATCC-CCL22), the origin of the producer cell line used for Pregsure© BVD, was cultured in DMEM (Gibco), supplemented with Glutamax™, 50 IU/ml Penicillin, 50 ug/ml Streptomycin and 10% FCS. A MDBK-derived cell line expressing the bovine herpes virus 1 (BHV-1) derived TAP inhibitor UL49.5 was used as a MDBK cell line with constitutively reduced MHC I expression and has previously been described by us^16^.

We employed the CRISPR/Cas9 system^47^,^48^ to generate β2-microglobulin (B2M) knockout MDBK cells. For this, we constructed a selectable lentiviral CRISPR/Cas vector which will be described elsewhere (manuscript in preparation). Briefly, we altered the lentiviral pSicoR vector (Addgene plasmid 11579, Tyler Jacks Lab, MIT) to express a human codon-optimized nuclear-localized Cas9 gene that was N-terminally fused to PuroR via a T2A ribosome-skipping sequence. This cassette was expressed from the human EF1A promoter. Additionally, we replaced the mouse U6 promoter with a human U6 promoter which drives expression of a guideRNA consisting of a bovine B2M-specific CRISPR RNA (target sequence GAAATTGATTTGCTGAAGAA) fused to the trans-activating CRISPR-RNA and a terminator sequence. MDBK cells were transiently transfected with this anti-B2M CRISPR/Cas9 vector using the Neon® transfection system (Invitrogen). After transfection, cells were stained for MHC I surface expression levels using PE-conjugated anti-MHC I mAb (W6/32, AbD Serotec) and sorted on a FACSAriaII (BD Biosciences). Cells were cloned by limited dilution and a clone showing no MHC I surface expression was selected for this study.

### Cloning and transfection of MHC class I

In order to transfect HEK-293 with MDBK-derived MHC I, full-length MHC I were amplified from cDNA using a mixture of forward primers Bov 21a/g and Bov 21-BSF together with reverse primers Bov 3 and Bov 3-BSF^46^, to account for known polymorphisms at the primer target sites, and were ligated into the pcDNA™ 3.1(+) vector (Invitrogen). 29 clones were sequenced using vector primers and internal primers Bov 9 and Bov 11^46^. Seqscape© was used to analyze the sequence files.

HEK-293 cells were transfected with representative clones of all different MDBK-derived MHC I alleles and a mock pcDNA™ 3.1(+) construct as a negative control using Lipofectamine® 2000 (Invitrogen) and were analyzed 36 hours after transfection.

### Flow cytometry and confocal microscopy

Mouse monoclonal antibodies (mAbs) used in this study include anti-MHC I (ILA88 unlabeled and FITC conjugated), anti-CD31-PE (CO.3E1D4), anti-CD41 (ILA164 unlabeled and PE conjugated) acquired from AbD serotec; anti-MHC I (PT85a), anti-integrin β1 (FW4-101) acquired from WSU Monoclonal Antibody Center; anti-B2M (B1.1G6) described by Liabeuf *et al*.^49^.

Cells were incubated with antibodies (Abs) diluted in PBS supplemented with 2% FCS and 0.01% Azide at 4 °C for 30 min. Serum and colostrum was diluted to a final concentration of 1:20 and Ab binding was detected using polyclonal biotinylated sheep anti-bovine IgG Abs (AbD Serotec) and Streptavidin-PE (Molecular Probes©). Binding of biotinylated IgG was directly detected using PE or A647 conjugated Streptavidin. Granulocytes and peripheral blood mononuclear cells (PBMC) were gated on forward and sideward scatter.. In all experiments appropriate isotype-matched control Abs were included. Flow cytometry experiments were performed on a FACSCanto™ (BD Biosciences) and data were analyzed using Flowjo software (TreeStar Inc.).

For confocal imaging of bone marrow cells, cells were stained as for flow cytometry. Next, cells were fixed in 4% formaldehyde and nuclei were counterstained with DAPI (Sigma Aldrich). Cells were spotted on microscope slides and images were acquired on a SPE-II confocal microscope (Leica).

### Immunoprecipitation and visualization/identification of precipitated protein

Cell surface proteins of MDBK cells or PBLk were labeled with EZ-Link™Sulfo-NHS-SS-Biotin (Thermo Scientific) according to manufacturer’s instructions. After biotinylation, cells were stained in serum diluted 1:20 and incubated at 4 °C for 45 min on a head-over-head roller. To assure binding of extracellular proteins only, cells were washed four times to wash away unbound and non-specifically bound Abs. Cells were lysed with ice-cold lysis buffer (1.0% Triton X-100, 20 mM MES, 100 mM NaCl, 30 mM Tris, pH 7.5) supplemented with protease inhibitors (cOmplete Protease Inhibitor Cocktails, Roche Life Sciences) at 4 °C for 30 min on a head-over-head roller. Supernatant, obtained after centrifugation (18,000 × g; 4 °C; 20 min), was incubated with Protein G coupled Dynabeads (Life Technologies) at 20 °C for 20 min on a head-over-head roller. After washing four times in lysis buffer, samples were boiled (95 °C; 5 min) in non-reducing lithium dodecyl sulphate sample buffer (Thermo Scientific). Beads were removed and samples were subjected to PAGE on Amersham ECL Gel 4–20% (GE Healthcare). Proteins were transferred to a nitrocellulose membrane (Protran, Whatman) using a semi-dry blotting system (Trans Blot Semi Dry, BioRad). The membrane was blocked with blocking reagent for ELISA (Roche). Biotinylated cell surface proteins were detected with alkaline phosphatase (AP) conjugated streptavidin (Sigma). MHC I was detected with anti-MHC I (ILA88) and AP conjugated goat anti-mouse IgG (Southern Biotech). Signals were developed with NBT/BCIP (Roche life sciences).

Non-biotinylated immunoprecipitated protein samples were processed in parallel and visualized using GelCode Blue Stain Reagent (Thermo Scientific) after PAGE. Bands corresponding to the bands found in the Western blots were excised as indicated in Fig. 4c and were sent for mass spectrometry analysis (Alphalyse Inc.). In short, protein samples were reduced and alkylated with iodoacetamide and trypsin digested, subjected to nano-liquid chromatography on an Ultimate 3000 system (Dionex) and subsequent MS/MS analysis on an Impact QTOF instrument (Bruker Maxis). The MS/MS spectra were analysed using Mascot (Matrix Science) and the UniProt and NCBI protein database were searched to identify protein matches.

### Antibody-dependent complement-mediated cell lysis

To assess antibody-dependent complement-mediated cell lysis, cells were incubated with Ab (monoclonal Abs anti-MHC I ILA88 & PT85a diluted to 0.5 μg/ml, serum of BNP or control dams diluted 1:100–1:400 for incubation with cell lines and 1:10 for incubation with PBLk) for 30 min at RT. Next, baby rabbit serum (Abd Serotec) was added to a final dilution of 1:10 and cells were incubated at 37 °C and 5% CO_2_ for 60 min. Following staining with DAPI, cell lysis was assessed using flow cytometry.

### Statistical analyses

Protein sequence homology was analysed using Mega6 to construct a phylogenetic tree using the Neighbor-Joining method and tree distances were calculated using the number of amino acid differences. Protein alignments were performed in Bioedit 7.2.5. The use of specific statistical tests (GraphPad Software) is mentioned in the figures legends. P values < 0.05 were considered significant.

## Additional Information

**How to cite this article**: Benedictus, L. *et al*. Pathogenicity of Bovine Neonatal Pancytopenia-associated vaccine-induced alloantibodies correlates with Major Histocompatibility Complex class I expression. *Sci. Rep*. **5**, 12748; doi: 10.1038/srep12748 (2015).

## Supplementary Material

Supplementary Information

## Figures and Tables

**Table 1:** Identification of proteins precipitated with sera from BNP dams.

Protein^a^	Calculated MW	Sequence coverage (%)	Number of peptides
Integrin alpha-3	117 kDa	19% (Sample 1)	82
		20% (Sample 2)	86
Integrin beta-1	91 kDa	21% (Sample 1)	86
		21% (Sample 2)	75

^a^Precipitated target Ags of BNP sera on B2M KO MDBK cells were identified using nano-liquid chromatography and subsequent mass spectrometry.
