# Supplementary material for: Pathogenicity of Bovine Neonatal Pancytopenia-associated vaccine-induced alloantibodies correlates with Major Histocompatibility Complex class I expression
Source: Sci Rep. 2015 Aug 3;5:12748. doi: 10.1038/srep12748 (PMC4522679; doi:10.1038/srep12748)
Supplement: Supplementary Information [file srep12748-s1.pdf]

Pathogenicity of Bovine Neonatal Pancytopenia-associated vaccine-induced alloantibodies correlates with Major Histocompatibility Complex class I expression

Lindert Benedictus, Rutger D. Luteijn, Henny Otten, Robert Jan Lebbink, Peter J.S. van Kooten, Emmanuel J.H.J. Wiertz, Victor P.M.G. Rutten, Ad P. Koets

Supplementary Table SI. BNP alloantibodies stain bovine peripheral blood mononuclear cells (PBMC) isolated from animals with a diverse MHC class I background.

MHC class I haplotype of PBMC donor[a]		BNP 1 (colostrum IgG)	BNP 2 (serum IgG)
A19v3	H5v2(UU)	459	988
A12 (UU)	A20v3UU	2478	1128
A14	A15v1	3663	2654
A20v3UU		4796	2505
A11		3611	3061
A14		331	3750
A15v1	UU1	2573	2066
A13	A10	4614	4220
A11		3291	3243
A10	H2	3189	2562
UU1	A18v2	2784	3393
A14		956	2071
A15v1		1926	923
A11	A20v3UU	4065	2408
A14		700	6407
A19v3		1708	1545
A15v1	A19v3	3275	1439
A19v3	UU7	2402	1542
A13	A12 (UU)	1800	1551
A15v1		2214	1455
A14	A19v3	910	1882
A13	A15v1	1296	2717

[a]PBMC isolated from MHC class I haplotyped animals (n=22) were stained by IgG isolated from the colostrum or serum of two dams and Ab binding was measured using flow cytometry. MHC class I haplotype of the IgG donors are A14 for BNP dam 1 and A12(UU) for BNP dam 2. GMFI = Geometric Mean Fluorescent Intensity. Bovine MHC class I haplotypes are based on Codner et al. (1) and Benedictus et al. (2). Haplotypes with a UU pre- or suffix are provisional haplotypes.

References

1. Codner, G. F., J. Birch, J. A. Hammond, and S. A. Ellis. 2012. Constraints on haplotype structure and variable gene frequencies suggest a functional hierarchy within cattle MHC class I. *Immunogenetics* 64: 435-445.
2. Benedictus, L., H. G. Otten, G. Van Schaik, W. G. J. Van Ginkel, H. C. M. Heuven, M. Nielen, V. P. M. G. Rutten, and A. P. Koets. 2014. Bovine Neonatal Pancytopenia is a heritable trait of the dam rather than the calf and correlates with the magnitude of vaccine induced maternal alloantibodies not the MHC haplotype. *Veterinary Research* 45: 129.

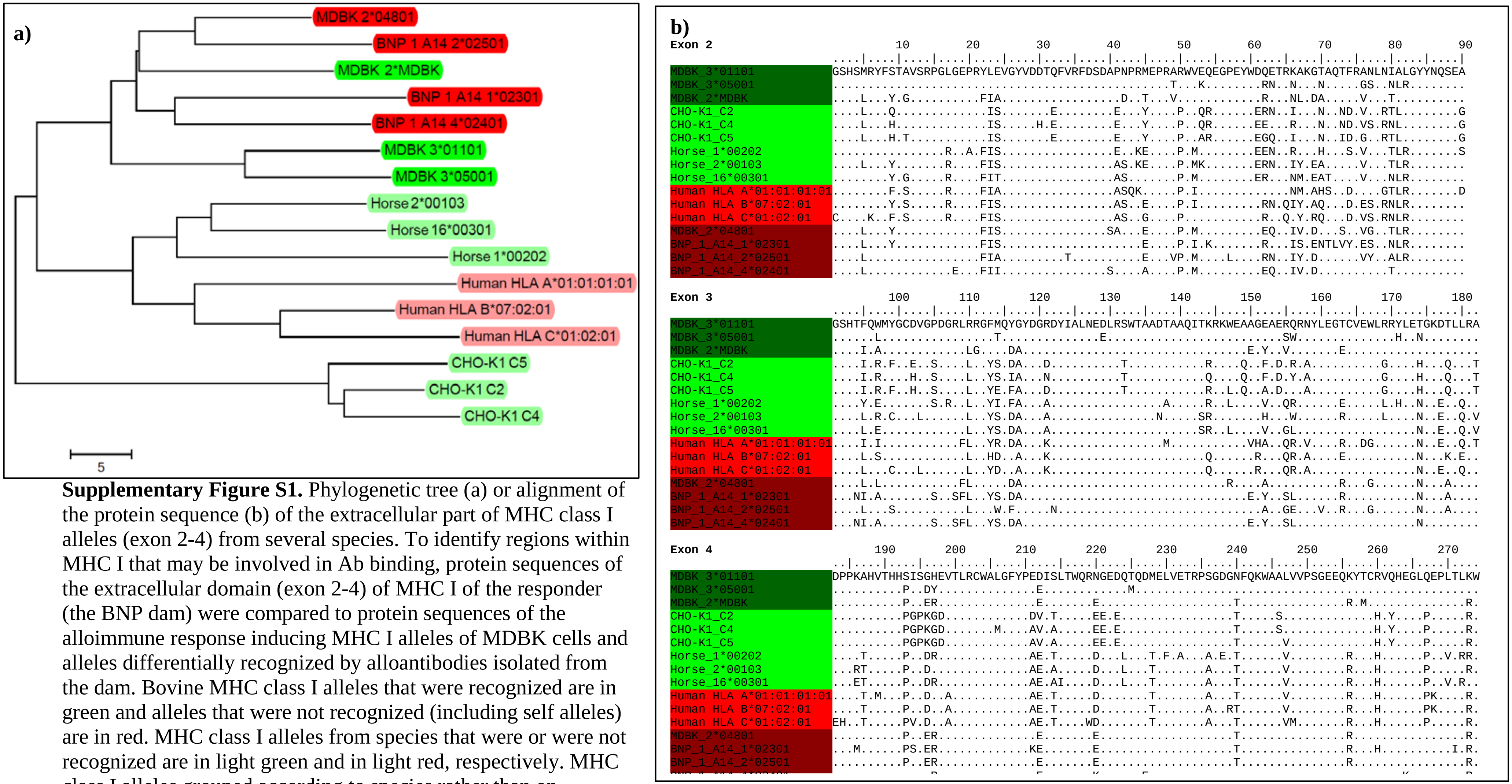

Supplementary Figure S1. Phylogenetic tree (a) or alignment of the protein sequence (b) of the extracellular part of MHC class I alleles (exon 2-4) from several species. To identify regions within MHC I that may be involved in Ab binding, protein sequences of the extracellular domain (exon 2-4) of MHC I of the responder (the BNP dam) were compared to protein sequences of the alloimmune response inducing MHC I alleles of MDBK cells and alleles differentially recognized by alloantibodies isolated from the dam. Bovine MHC class I alleles that were recognized are in green and alleles that were not recognized (including self alleles) are in red. MHC class I alleles from species that were or were not recognized are in light green and in light red, respectively. MHC class I alleles grouped according to species rather than on recognition by serum alloantibodies of BNP dam 1 and protein alignments revealed no apparent relation between Ab binding and differences in protein sequence between responder, alloimmune response inducing and differentially recognized MHC class I alleles. (A) The phylogenetic tree was constructed using the Neighbor-Joining method and tree distances were calculated using the number of amino acid differences. (B) All sequences were compared to the first allele and dots represent identities. MHC class I alleles that were differentially recognized by alloantibodies from BNP dam 1 were based on the information from figure 1 and 2. For human, horse and CHO-K1 sequences it was not known which specific alleles were recognized. Horse alleles were selected from the most common classical MHC class I genes 1, 2 and 16 (1). For humans representative alleles of the classical HLA-A, HLA-B and HLA-C gene were selected from the IMGT/HLA Database (http://www.ebi.ac.uk/ipd/imgt/hla/). For CHO-K1 the expressed classical MHC class I alleles were added (2).

References

1. Tallmadge, R. L., J. A. Campbell, D. C. Miller, and D. F. Antczak. 2010. Analysis of MHC class I genes across horse MHC haplotypes. *Immunogenetics* 62: 159-172.

2. Furukawa, H., K. Iizuka, J. Poursine-Laurent, N. Shastri, and W. M. Yokoyama. 2002. A ligand for the murine NK activation receptor Ly-49D: Activation of tolerized NK cells from beta(2)-microglobulin-deficient mice. *Journal of Immunology* 169: 126-136.

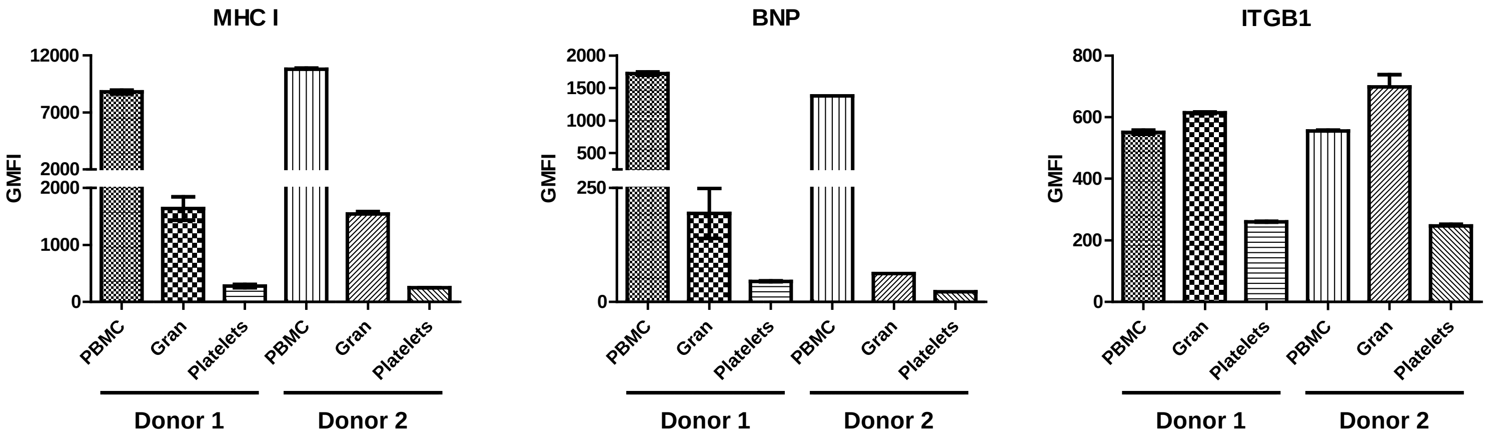

Supplementary Figure S2.

Expression of MHC class I, Integrin beta-1 and BNP Ab binding on peripheral blood leukocytes and platelets. Peripheral blood leukocytes and platelets (n=2, donor 1 & 2) were stained with mAbs against MHC class I (MHC I), Integrin beta-1 (ITGB1) or with BNP Abs. Expression/Ab binding was measured using flow cytometry. GMFI = Geometric Mean Fluorescent Intensity. PBMC = peripheral blood mononuclear cells. Gran = granulocytes.

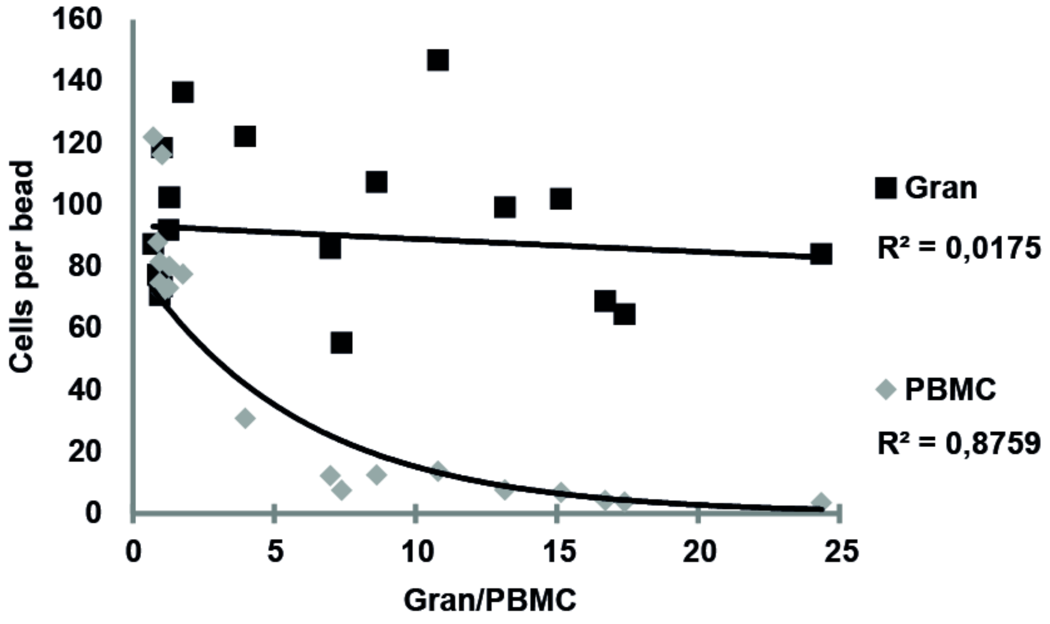

Supplementary Figure S3.

Peripheral blood leukocytes from calves were incubated with fluorescent microspheres during the complement lysis assay as an internal control. The number of cells per bead (Granulocytes (Gran) or PBMC) are plotted against the proportion of Gran to PBMC for several serum and colostrum samples from Pregsure© BVD vaccinated BNP and non-BNP dams and unvaccinated dams. The number of cells per bead are expressed relative to the number of cells per bead for the medium control. An exponential trend line between cells per bead and the proportion Gran to PBMC is plotted and the R2 for the trend line is shown. Data are representative for three different animals.
